# Evaluation of splanchnic oximetry, Doppler flow velocimetry in the superior mesenteric artery and feeding tolerance in very low birth weight IUGR and non-IUGR infants receiving bolus *versus* continuous enteral nutrition

**DOI:** 10.1186/1471-2431-12-106

**Published:** 2012-07-24

**Authors:** Valentina Bozzetti, Giuseppe Paterlini, Valeria Meroni, Paola DeLorenzo, Diego Gazzolo, Frank Van Bel, Gerard HA Visser, MariaGrazia Valsecchi, Paolo E Tagliabue

**Affiliations:** 1 Department of Neonatal Intensive Care, MBBM Foundation, San Gerardo Hospital, via Pergolesi 33, 20900, Monza, Italy; 2 Department of Clinical and Preventive Medicine, Center of Biostatistics for Clinical Epidemiology, University of Milano-Bicocca, Monza, Italy; 3 Department of Pediatrics, University of Milano-Bicocca, S. Gerardo Hospital, Monza, Italy; 4 Department of Maternal, Fetal and Neonatal Medicine, C. Arrigo Children’s Hospital, Alessandria, Italy; 5 Department of Neonatology, Wilhelmina Children’s Hospital, AB, Utrecht, Netherlands

**Keywords:** Feeding tolerance, Near infrared spectroscopy, Minimal enteral feeding, Enteral nutrition, Parenteral nutrition, Intra-uterine growth restriction, Near infrared spectroscopy, Mesenteric artery Doppler, *Bolus* nutrition, Intermittent nutrition

## Abstract

**Background:**

IUGR infants are thought to have impaired gut function after birth, which may result in intestinal disturbances, ranging from temporary intolerance to the enteral feeding to full-blown NEC.

In literature there is no consensus regarding the impact of enteral feeding on intestinal blood flow and hence regarding the best regimen and the best rate of delivering the enteral nutrition.

**Methods/design:**

This is a randomized, non-pharmacological, single-center, cross-over study including 20 VLBW infants.

Inclusion criteria

* Weight at birth ranging: 700–1501 grams

* Gestational age up to 25 weeks and 6 days

* Written informed consent from parents or guardians

Exclusion criteria

* Major congenital abnormality

* Patients enrolled in other trials

* Significant multi-organ failure prior to trial entry

* Pre-existing cutaneous disease not allowing the placement of the NIRS’ probe

In the first 24 hours of life, between the 48^th^ and 72^nd^ hours of life, and during Minimal Enteral Feeding, all infants’ intestinal perfusion will be evaluated with NIRS and a Doppler of the superior mesenteric artery will be executed.

At the achievement of an enteral intake of 100 mL/Kg/day the patients (IUGR and NON IUGR separately) will be randomized in 2 groups: Group A (n=10) will receive a feed by *bolus* (in 10 minutes); then, after at least 3 hours, they will receive the same amount of formula administered in 3 hours. Group B (n=10) will receive a feed administered in 3 hours followed by a *bolus* administration of the same amount of formula (in 10 minutes) after at least 3 hours.

On the randomization day intestinal and cerebral regional oximetry will be measured via NIRS. Intestinal and celebral oximetry will be measured before the feed and 30 minutes after the feed by *bolus* during the 3 hours nutrition the measurements will be performed before the feed, 30 minutes from the start of the nutrition and 30 minutes after the end of the gavage. An evaluation of blood flow velocity of the superior mesenteric artery will be performed meanwhile. The infants of the Group A will be fed with continuous nutrition until the achievement of full enteral feeding. The infants of the Group B will be fed by *bolus* until the achievement of full enteral feeding.

**Discussion:**

Evaluations of intestinal oximetry and superior mesenteric artery blood flow after the feed may help in differentiating how the feeding regimen alters the splanchnic blood flow and oxygenation and if the changes induced by feeding are different in IUGR versus NON IUGR infants.

**Trial registration number:**

NCT01341236

## Background

Intra-uterine growth restriction (IUGR) caused by placental insufficiency is characterized by blood flow redistribution to the vital organs (brain, myocardium, and adrenal glands), while other organs, including the gastrointestinal tract, are deprived from sufficient blood flow. As a consequence of gut ischemia/hypoxia, IUGR infants are thought to have impaired gut function after birth, which may result in intestinal disturbances, ranging from temporary intolerance to the enteral feeding to full-blown NEC.

In literature, however, there is no consensus regarding the impact of enteral feeding on intestinal blood flow and hence regarding the best regimen and the best rate of delivering the enteral nutrition.

Doppler ultrasonography is the method currently used for the clinical assessment of velocity of superior mesenteric artery blood flow [1]. Blood flows parameters in the superior mesenteric artery (SMA) change with vasoconstriction or vasodilatation of the intestinal vascular bed. Prenatal utero-placental insufficiency with chronic fetal hypoxia can lead to foetal growth retardation with a redistribution of blood flow favouring the cerebral circulation and reducing mesenteric perfusion [2]. This underlines the importance of chronic or acute hypoxia as the most intensively studied condition associated with disturbances of intestinal motility.

The possible association between the increase in blood flow velocity and change in tissue oxygenation is expected. Greater understanding of the rate of oxygen delivery and uptake in sick preterm infants undergoing intensive care is an important aim of neonatal medicine.

The assessment of adequate perfusion in very low birth weight infants is commonly based on clinical parameters, as well as invasive measures requiring central venous and/or arterial catheter access with well established associated risks. Additionally, most of these data are acquired intermittently, and thus may only represent a delayed picture of oxygen delivery and consumption.

Near-infrared spectroscopy (NIRS) is a continuous, non-invasive, real-time and portable techinique, which can be used to measure oxygenation in living tissue [3].

In 1985, Brazy and Lewis [4] reported the first pediatric application of NIRS monitoring of cerebral oxygenation in sick preterm infants. Since then the list of publications on NIRS for hemodynamic and oxygenation assessment in children and adults has rapidly expanded [5,6].

The technological background of NIRS technology has been reviewed in detail [7]. The main principle upon which NIRS device relies is the fact that most biological tissues, other than haemoglobin and cytochrome oxidase, are relatively transparent to infrared light in the range closest to the visual spectrum (700–1000 nanometers), and that the absorbance spectrum of the haemoglobin depends on its oxygenation status (deoxygenated haemoglobin absorbs more red light and less infrared light than oxygenated haemoglobin). All devices emit lights at wavelengths within the above mentioned spectrum and analyze photons returning to the transducer. Because the change in the intensity of the reflected light is dependent upon the oxyhemoglobin to deoxyhemoglobin ratio, oxyhemoglobin saturation can be derived [8]. There are many different NIRS devices available. We use the INVOS cerebral oximeter (Somanetics Corporation, Troy, Michigan USA) that is FDA approved for adult and pediatric use including infants [9].

NIRS has been used to monitor oxygenation of the brain in neonates by measuring the ratio of oxygenated to deoxygenated hemoglobin (termed “tissue oxygenation index”, TOI) [10]. NIRS has been reported to be useful in detecting changes in splanchnic oxygen delivery and predicting splanchnic ischemia in neonates by measuring the ratio of splanchnic to cerebral TOI, the cerebrosplanchnic oxygenation ratio (CSOR). Splanchnic oxygenation is compared with brain oxygenation as a reference, because under most of physiological conditions cerebral blood flow autoregulation minimizes changes in brain oxygenation during events affecting splanchnic perfusion [11].

A significant concern with the application of NIRS to the abdomen is the possibility of movement of the gut within the abdomen and also movement produced by peristalsis of the gut wall. These two movements can alter the scattering path of the near infrared light, resulting in absorption changes, which would swamp the signal of interest [12,13]. However TOI now offers a method of comparing the haemoglobin redox status within the splanchnic circulation, which is not path-length-dependent because it provides a simultaneous ratio of oxyhaemoglobin to deoxyhaemoglobin. Finally, by measuring the TOI of the brain, which is preferentially autoregulated with the splanchnic region under most physiological conditions, the resultant CSOR ratio gives absolute values, which allow comparison between individual patients. CSOR had a 90% (56-100%) sensitivity to detect splanchnic ischaemia in neonates [14].

Regional tissue oxygenation of some other vascular beds and its clinical relevance is under review in extremely low birth weight infants [15].

In an effort to decrease the risk for development of NEC in preterm infants, enteral nutrition is often delayed when the neonate shows signs of feeding intolerance. However, enteral fasting can predispose a neonate to impaired intestinal growth, mucosal atrophy, intestinal barrier dysfunction, decreased digestive and absorptive capacity, increased colonization with pathogenic bacteria, and systemic inflammation. In addition, enteral fasting can prolong the time to establish full enteral feeding and the length of hospital stay [16]. Consequently, minimal enteral feeding (MEF) in combination with parenteral nutrition (PN) is often employed to alleviate the side effects of enteral fasting in premature infants. MEF is thought to promote intestinal motility, to maintain the intestinal barrier, to stimulate the development of “good” microflora, and to reduce infections.

Tube feeding is necessary for most premature infants less than 1500 grams because of their inability to coordinate sucking, swallowing, and breathing and the risk of aspiration. The conventional tube feeding method is intermittent bolus gavage feeding, where a prescribed volume of milk is given over a short period of time, usually over 10 to 20 minutes by gravity. Some clinicians prefer the continuous nasogastric route to feed premature infants less than 1500 grams birth weight, although, in practice, intermittent bolus gavage feeding is the method more commonly used [17,18]. In our Unit VLBW infants are fed by boluses, although they are often empirically switched to the continuous infusion method without an established rationale. Theoretical risks and benefits of both continuous nasogastric milk feeding and intermittent bolus milk feeding have been proposed. Continuous nasogastric feedings may improve energy efficiency (by increasing energy absorbed and decreasing energy expenditure), reduce feeding intolerance, improve nutrient absorption, and improve growth. However, continuous infusion of milk into the gastrointestinal tract could alter the cyclical pattern of release of gastrointestinal tract hormones, which might affect metabolic homeostasis, and growth. Milk feedings given by bolus gavage method are thought to be more physiologic because they promote the cyclical surges of gastrointestinal tract hormones normally seen in healthy term infants. On the other hand, functional limitations of the premature infant’s gastrointestinal system such as delayed gastric emptying or intestinal transit could hinder the premature infant’s ability to handle bolus milk feeds, resulting in feeding intolerance.

Additionally, this feeding regimen that alternates between periods of feeding and fasting may challenge the premature infant’s ability to maintain metabolic homeostasis and, therefore, decrease growth. There is still a debate about which is the best feeding regimen in order to prevent episodes of feeding intolerance and to promote a better growth.

### Aims of the study

Primary aim of this study:

1. To evaluate the changes in the intestinal perfusion and oximetry determined by feeding in VLBW infants fed by 3 hours nasogastric nutrition (CN) *versus* infants fed by bolus nutrition (BN).

Secondary aims of the study:

2. To compare if changes in the intestinal perfusion and oximetry induced by feeding are different between IUGR and NON-IUGR infants.

3. To compare growth and nutritional status of the 2 groups by randomized arm.

4. To test if changes in intestinal oximetry and perfusion can be reliable predictors of feeding intolerance (days necessary to achieve full enteral feeding)*.*

### Endpoints

The endpoint for the primary aim will be the cross-over difference of CSOR values, measured with NIRS before and at the end of the randomized feeds.

Aims 1 to 4 will be pursued analysing the following endpoints:

· The cross-over difference of CSOR values and of rSO2s values (i.e. the splanchnic saturation slope) in IUGR and non-IUGR infants.

· Growth and nutritional status will be measured by weight, length and head circumference. Main comparison will be between measures at randomization and at achievement of full enteral feeding. The growth at 28 days of life and at 36 weeks of gestational age will also be compared with the appropriate standard growth curves.

· The difference in the CSOR values pre- and post- feeding will be compared with the baseline CSOR value and the baseline Doppler flow velocimetry (both measured within the first 72 hours of life)*.*

· Comparison of the time needed to reach full enteral feeding (i.e. days from randomization till enteral intake of 160 ml/kg/day of formula or fortified human milk) by randomized arm;

## Methods and design

This is a randomized, non-pharmacological, single-center, cross-over study. Duration of the study: 24 months. The study takes place in the Neonatal Intensive Care Unit.

This study aims at recruiting about twenty very low birth weight infants, either IUGR or NON-IUGR.

### Inclusion criteria

· Weight at birth ranging: 700–1501 grams;

· Gestational age up to 25 weeks and 6 days;

· Written informed consent from parents or guardians.

### Exclusion criteria

· Major congenital abnormalities (severe heart or cerebral disease, chromosomopathies, severe renal malformations, any malformation or disease of the gastroenteric tract)

· Patients enrolled in other trials

· Significant multi-organ failure prior to trial entry (perinatal asphyxia with renal, cardiac or cerebral impairment, DIC)

· Pre-existing cutaneous disease not allowing the placement of the probe

### Eligibility to randomization

Infants who fulfill the following requirements are eligible to randomization:

· achievement of at least 100 mL/Kg/day of enteral nutrition

· adequate ventilation i.e. infants who are not intubated and not in-cPAP with a FiO2≥50% at the achievement of 100 mL/Kg/day of enteral nutrition;

· no NEC;

· Written informed consent from parents or guardians.

#### Stratification

Randomization will be stratified in two groups: IUGR infants (approximately 10 children) and NON-IUGR infants (approximately 10 children).

At the achievement of an enteral intake of 100 mL/Kg/day the patients (IUGR and NON IUGR separately) will be randomized in 2 groups: Group A (n=10) will start with bolus administration of nutrition (in 10 minutes); then, after at least 3 hours, they will be fed by the same amount of feed administered as continuous nutrition for 3 hours; Group B (n=10) will start with continuous administration of nutrition for 3 hours followed by a bolus administration of the same amount of feed (in 10 minutes) after at least 3 hours.

The infants of the Group A will be fed with continuous nutrition until the achievement of full enteral feeding (enteral intake of 160 mL/Kg). The infants of the Group B will be fed with 7 or 8 boli/d until the achievement of full enteral feeding.

If, due to clinical instability (i.e. desaturation episodes or bradicardia) during the feeding, a change occurs in the modality of feeding, the results will be analyzed according to the “intention to treat” or the “by treatment” analysis.

All the patients will undergo a baseline evaluation in the first 72 hours of life including: cerebral ultrasound, cardiac ultrasound and abdominal ultrasound.

After birth, in the first 24 hours of life, and in the transitional period, between the 48^th^ and 72^nd^ hours of life, all infants’ intestinal perfusion will be evaluated with NIRS and the echocolordoppler (evaluation of the superior mesenteric artery blood flow) will be performed. The evaluations with NIRS and with the echocolordoppler will be performed under condition of clinical stability (absence of arterial desaturation or instability of cardiocirculatory parameters). Data from NIRS will be collected for 3 hours. Other anamnestic and clinical data will be collected (gestational age, umbilical arterial pH, race, obstetrical anamnesis, mode of delivery, umbilical arterial and venous catheters, patent ductus arteriosus, respiratory distress syndrome, mechanical ventilation, episodes of clinical sepsis). All the infants will be at nothing per os (npo) at the moment of the first two evaluations.

According to our protocol enteral nutrition will start after the 72^nd^ hour of life as minimal enteral feeding (MEF), intake less than 20 mL/Kg/day of enteral feeding will be administered. The increase will be by 20 mL/Kg/day if enteral nutrition is well tolerated. All the infants, according to our standardized protocol, will start parenteral nutrition on the first day of life. The infants will be fed with human milk, if available, (human milk will be fortified at an achievement of an enteral intake of 100 ml/Kg/d), or with a preterm formula (75–80 Kcal/100 mL). The nutrition will be administered via the nasogastric route.

On the randomization day intestinal and cerebral regional oximetry will be measured via near infrared spectroscopy (NIRS) (INVOS- 5100 C) sensors placed over the abdomen (splanchnic bed) and on the forehead (cerebral bed). Recording of the tissues oximetry will start 30 minutes before the feed and will stop 30 minutes after the end of the nutrition. Intestinal and celebral oximetry will be measured before the feed (B_0_) and 30 minutes after the feed (B_1_) by bolus; during the continuous nutrition the measurements will be performed before (C_0_), 30 minutes from the start of the nutrition (C_1_) and 30 minutes after the end of the gavage (C_2_).

The TOI will be obtained simultaneously at the two locations. The TOI measurement recordings will be obtained for 3 minutes from both the head and the abdomen. Those measurements will be combined as a ratio of abdominal TOI over brain TOI to produce a CSOR (TOI_abdomen_/TOI_brain_). Arterial haemoglobin oxygen saturation, measured by pulse oximetry, will be recorded during the NIRS tracing. NIRS tracing will be used only in the absence of desaturation (< 85% arterial saturation).

Capillar haemoglobin concentration will be measured on the day of the evaluation.

An evaluation of blood flow velocity of the superior mesenteric artery (peak sistolic and end-diastolic velocity, mean velocity, and pulsatility index) will be performed meanwhile. To achieve imaging of the SMA the transducer will be placed on the mid-abdomen above the umbilicus. The SMA will be identified at its emergency from the aorta and sample volume will be placed a few millimetres from its origin. Neonatologists with the same expertise and manual ability will perform the ecocholordoppler evaluations. The evaluations and the measurements will be performed on 5 contiguous homogeneous waves.

Measurements of body weight, length and head circumference will be performed at predefined times: at birth, at the beginning of MEF, on the randomization day, at the achievement of full enteral feeding, at 28 days of life and at 36 weeks of gestational age.

Parameters of feeding tolerance are those routinary used in the NICU (maximum gastric residual and residual appearance). The enteral nutrition will be discontinued or carried on according to our feeding protocol.

The follow-up will end at the achievement of a full enteral feeding, enteral intake of 160 mL/Kg/die or at 28 days of life, whichever occurs later. Patients may drop-out before this intended end because of the following withdrawal causes:

Withdrawal of the consent by the relatives

Severe skin reaction due to the skin probe

Transferral to another hospital

Death

After study end, patients will be fed according to the standard protocol of our Unit.

During the study, recommended diagnostic and therapeutic procedures are those usually performed as standard care in our Unit.

No new special measure for safety is planned since all the diagnostic and therapeutic procedures are part of the standard approach to the VLBW infant in our Unit.

### Statistical considerations

#### Randomization

Randomized interventions are described in Section ‘Methods and Design’. This section summarizes procedures and methodology, which will be adopted**.**

Patients eligible to randomization: all VLBW infants enrolled and eligible to the study, either IUGR or non-IUGR, who fulfil the following requirements:

1. achievement of 100 mL/kg/day of enteral nutrition;

2. adequate ventilation i.e. infants who are not intubated and not in-cPAP with a FiO2≥50% at the achievement of 100 mL/Kg/day of enteral nutrition;

3. no NEC;

4. Informed consent from parents or guardians.

When to randomize:

a. * After *achievement of 100 mL/kg/day of enteral nutrition in presence of adequate ventilation (see point 2 above)

Modalities of randomization:

b. Logistics: investigators will ask for randomization by access to a specific software which will provide the assigned arm, after check on the eligibility criteria.

c. Methodology: randomization will be stratified (IUGR vs non-IUGR) and by blocks: the random assignment will be produced by an automatic procedure Ranlist [19] and will be based on random permuted blocks of small size, given the limited number of infants expected in each stratum.

**Randomization refusals:** if parents or guardians do not agree with randomization, patients will be excluded from the study and should be fed according to nutrition procedure regarded as the most appropriate for the child based on the Unit standard policy.

#### Statistical analysis

The primary aim of the study is to compare the impact on intestinal perfusion of 2 different nutrition modalities, bolus (BN, Group A) versus a 3-hour nasogastric nutrition (CN, Group B). The impact on intestinal perfusion is defined as the difference between pre- and post-prandial CSOR values measured with NIRS 30 minutes before feeding and at the expected peak after feeding (3 hours in CN and 30 minutes in BN). Pre- and post-prandial CSOR values will be calculated as the mean of 5-minute NIRS evaluations about the intended time-point (e.g. for baseline CSOR measurement, 2.5 minutes before and 2.5 minutes after the 30th minute before start of the randomized feed). Given that the study is a cross-over study, each infant will be evaluated both for CN and BN and the cross-over CSOR difference will be the primary endpoint.

As a secondary analysis, the cross-over difference of CSOR values as well as that of SO2s values (i.e. the splanchnic saturation slope) will be analysed comparing intra-uterine growth (IUGR vs. non-IUGR).

The impact of the 2 nutrition modalities on CSOR will also be studied in a multivariable context, adjusting for factors including the baseline CSOR value, the baseline Doppler flow velocimetry (both measured within the first 72 hours of life) and the intra-uterine growth (IUGR vs. non-IUGR).

Other secondary analyses include:

· Comparison of the time needed to reach full enteral feeding (i.e. days from randomization till enteral intake of 160 mL/Kg/day of formula or human milk) by randomized arm;

· Comparison of growth as measured by weight, length and head circumference by randomized arm. Main comparison will be between measures at randomization and at achievement of full enteral feeding. The growth at 28 days of life will also be compared with the appropriate standard growth curves.

The primary end-point analysis as well as all secondary analyses regarding randomized patients will be based on the ITT (intention-to-treat) principle. Comparisons of the two arms accounting for deviations from the assigned arm (analyses by “performed treatment”) will also be added to this main analysis. The primary endpoint will be analyzed with a two-tailed matched pairs t-test, after checking of appropriateness of assumptions. In particular, checking will concern the normality assumption, since period effect and cross-over effect appear both very unlikely in this study. Should normality be not adequate, non-parametric test such as the Wilcoxon signed ranks test will be applied [20]. An appropriate generalized linear model will be used to investigate the relationship between the CSOR cross-over difference and the candidate covariates. Should any raw CSOR observations be missing (e.g. NIRS instrument failed to measure or to save measurements), their estimation via imputation or other appropriate methods will be taken into account. The comparison of growth variables by randomized arm will be performed with either the two-tailed t-test or a non-parametric alternative (e.g. Mann–Whitney test), should the normality assumption be not reasonable. The time from randomization till achievement of the full enteral feeding will be analysed with the Kaplan-Meier estimator, in which full enteral feeding as defined in the protocol will be considered as the sole ‘event’, while censoring will occur for withdrawal for any cause. Standard errors will be computed according to Greenwood formula and the log-rank test will be used for univariate comparisons.

### Sample size

This single-center study will recruit patients for 2 years (24 months) since official opening. The expected accrual is about 20 VLBW infants, either IUGR or non-IUGR. The power calculations below are based on a two-tailed t-test with I-type error α=0.05 and show the power that the 20-patient study will achieve under various differences in CSOR from the ‘baseline’ impact observed under BN. On the basis of previous studies [21] this is expected to be 0.10 (mean of CSOR pre-post prandial difference), while various hypothesis are made about its standard deviation, σ_CSOR_

### Sample size: 20 infants

The power calculations in different scenarios show that with 20 patients the study will achieve a good power in presence of a cross-over difference of at least 0.07 (Table 1).

**Table 1:** Impact on CSOR under BN=0.10

**σ**_**CSOR**_	**CSOR cross-over difference**	**Power**
0.10	0.07	84%
0.15	0.10	80%
0.20	0.13	78%

### Data collection

The case report form (CRF) attached to the protocol describes the data needed for each patient (see Additional file 1). A specific database will be set up which will capture the data produced by the NIRS instrument and automatically saved in an exportable file and the demographic and clinical data routinely available in the electronic clinical chart used by the center. Other variables, collected *ad hoc* for this study, will be entered in the same database by the Trial Data Center.

## Discussion

This is an explorative study. Evaluations of intestinal oximetry and superior mesenteric artery blood flow after the feed may help in differentiating how the feeding regimen alters the splanchnic blood flow and oxygenation and if the changes induced by feeding are different in IUGR versus NON IUGR infants.

We postulate that TOI will be altered in the vascular bed when blood flow is decreased; the resultant acidosis in poorly perfused tissues may also further increase the dissociation of oxygen from haemoglobin and increase the portion of reduced haemoglobin detected with NIRS. This condition may be present in IUGR infants and this condition may alter feeding tolerance thus requiring a longer time to achieve full enteral feeding and increasing the episodes of feeding intolerance.

Gut perfusion may depend on the way of administering feeding, bolus or continuous nutrition, so this study may suggest which is the best way to feed VLBW infants.

### Legal and ethical requirements

Direct access to data/original documents.

I/We hereby declare that the experimenter will grant examination, revision of the IRB/IEC, and the inspection by the competent authority through direct access to data/original documents.

### ’Liability

#### Legality of the study

The study will be conducted in conformity with the laws in force.

Protection of the patient’s personal data.

Participants’ personal data and the study results will be treated confidentially according to DL 30/06/2003 n.196.

#### Informed consent

Parents (or people with parental authority) of eligible patients will be informed and provided with details by any of the assigned doctors.

#### Ethics committee approval

The study received approval of the Ethics Committee.

#### Helsinki declaration

The study will be conducted in conformity with principles and regulations of the Helsinki Declaration and its amendments.

#### Quality assurance and quality control

Reference to the guidelines for Clinic Good Practice (CPMP/ICH/135/95).

The Center of Biostatistics for Clinical Epidemiology, University of Milano-Bicocca will analyze the outcomes of the study.

Regulation of the data promulgation.

The final results of the study will be published even in case of non attainment of the goals. The publication will refer to the “CONSORT Statement” [22,23] and will include complete analyses on security. Data of the study shall be published or reproduced upon Trial Steering Committee notice.

## Abbreviations

NEC, Necrotizing enterocolitis; NIRS, Near infrared spectroscopy; MEF, Minimal enteral feeding; EN, Enteral nutrition; NPT, Total parenteral nutrition; VLBW, Very low birth weight; IUGR, Intra-uterine growth restriction; TOI, Tissue oxygenation index; CSOR, Cerebrosplanchnic oxygenation ratio; CN, Continuous nutrition; BN, Bolus nutrition; NPO, Nothing per os; PSV, Peak sistolic velocity; EDV, End-diastolic velocity; Vmean, Mean velocity; RI, Resistance index; SMA, Superior mesenteric artery.

## Competing interests

The authors declare that they have no competing interests.

## Authors’ contributions

VB, VM and GP made substantial contributions to the design of the study and the acquisition of data. MGV and PDeL have been involved in drafting the protocol and defined the statistical procedures. DG, GHV, FVanB and PET revised critically the protocol. All the authors gave final approval to the ultimate version being published.

This study has received, on September 22th 2011, the ethical approval by the scientific and ethical committee of San Gerardo Scientific Institute of Monza, Italy.

This study has not received funding.

## Pre-publication history

The pre-publication history for this paper can be accessed here:

http://www.biomedcentral.com/1471-2431/12/106/prepub

## Supplementary Material

Additional file 1 Case report form. Click here for file
