# Supplementary material for: Evaluation of splanchnic oximetry, Doppler flow velocimetry in the superior mesenteric artery and feeding tolerance in very low birth weight IUGR and non-IUGR infants receiving bolus versus continuous enteral nutrition
Source: BMC Pediatr. 2012 Jul 24;12:106. doi: 10.1186/1471-2431-12-106 (PMC3447641; doi:10.1186/1471-2431-12-106)
Supplement: Additional file 1 — Case report form. [file 1471-2431-12-106-S1.pdf]

SPLANCHNIC OXIMETRY IN VLBW | No.1

REGISTRATION

ID: |_|_|_|_|_|_| Last name initials: |_|_|_| First name initials: |_|_|_| Gender: M F

Date of birth: |_|_|_|_|_|_| (d d m m y y) Race: |_|_| CODES: 1=Mediterranean Europe; 2=Central Europe; 3=Eastern Europe; 4=North Africa; 5=South Africa; 6=Central Africa; 7=Middle East 8=Far East; 9=North America; 10=Central America; 11=South America

ELIGIBILITY AT BIRTH

Inclusion criteria:

Was consent obtained? |_| CODES: 1=YES; 2=NO

Weight at birth |_|_|_|_| g GA |_|_|.|_| (w w d)

Exclusion criteria (see protocol for definitions):

Major congenital abnormality? |_| MOF? |_| CODES (for both): 1=YES; 2=NO; 9=NK

Pre-existing cutaneous disease? |_| CODES: 1=YES; 2=NO

Is the patient eligible according to protocol? |_| CODES: 1=YES; 2=NO; 6=Not evaluable; 9=NK

STRATIFICATION

IUGR: |_| CODES: 1=YES; 2=NO

RANDOMIZATION

Date of achievement of 100 ml/kg/day |_|_|_|_|_|_| (d d m m y y)

Ventilation at achievement of 100 ml/kg/day |_| CODES: 1=NO n-cPAP; 2=n-cPAP< 50%; 3= n-cPAP ≥ 50%; 4= Intubation

Do you plan to randomize? |_| CODES: 1=YES; 2=NO

IF YES: Date of randomization |_|_|_|_|_|_| (d d m m y y)

Random assigned |_| CODES: 1=Bolus+continuous; 2=Continuous+bolus

Random administered |_| CODES: 1=Bolus+continuous; 2=Continuous+bolus

If random administered is different from random assigned, specify reason |_| CODES: 1=Clinical decision; 2=Parents' refusal of consent; 3=Error; 4=Other; 9=Not known

If Other, specify: ______________________

IF NO: Cause of NO RANDOM |_| CODES: 1=No achievment of 100ml/kg/day; 2=Intubation or n-cPAP≥ 50%; 3=NEC; 4=Transfer to other istitution;5=Parents' refusal of consent ; 6=Error;7=Other;8=Death;9=Not known

If Other, specify: ______________________

______________________ ______________________ ______________________

Name of physician | Date (dd-mm-yy) | Signature of physician

SPLANCHNIC OXIMETRY IN VLBW | No.2

ID: |_|_|_|_|_|_| Last name initials: |_|_|_| First name initials: |_|_|_|

BEFORE BIRTH

Mother: CODES (FOR ALL) : 1=YES; 2=NO; 9=NK

PROM |_| Pre eclampsia |_| Chorionamnionitis |_| Placental abruption |_|

Antibiothic therapy |_| Indomethacin |_| Antepartum steroids |_|

Fetus:

Doppler |_| CODES : 1=Normal; 2=Pathological; 3=Absent; 4=Reverse; 9=Not known

AT BIRTH

Type of delivery |_| CODES: 1=PE; 2=TC funicolar pH |_|_|_| IA 5' |_|_|

AFTER BIRTH

Heart US |_| Cerebral US |_| Abdominal US |_| CODES (FOR ALL) : 1=Normal; 2=Pathological; 9= Not done

UAC |_| CODES: 1=YES; 2=NO

IF <u>YES</u>: Date of placement |_|_|_|_|_|_| (d d m m y y) Date of removal |_|_|_|_|_|_| (d d m m y y)

Nutrition:

UVC |_| CODES: 1=YES; 2=NO

IF <u>YES</u>: 1st placem. Date of placement |_|_|_|_|_|_| (d d m m y y) Date of removal |_|_|_|_|_|_| (d d m m y y)

CVC |_| CODES: 1=YES; 2=NO

IF <u>YES</u>: 1st placem. Date of placement |_|_|_|_|_|_| (d d m m y y) Date of removal |_|_|_|_|_|_| (d d m m y y)

2nd placem. Date of placement |_|_|_|_|_|_| (d d m m y y) Date of removal |_|_|_|_|_|_| (d d m m y y)

TPN 1st episode Date of start |_|_|_|_|_|_| (d d m m y y) Date of end |_|_|_|_|_|_| (d d m m y y)

2nd episode Date of start |_|_|_|_|_|_| (d d m m y y) Date of end |_|_|_|_|_|_| (d d m m y y)

Name of physician ____________ Date (dd-mm-yy) ____________ Signature of physician ____________

SPLANCHNIC OXIMETRY IN VLBW | No.3

ID: | | | | | | | Last name initials: | | | First name initials: | | |

OXIMETRY AND PERFUSION

		0-24 h	48-72 h	MEF 12 ml/kg	AT RANDOMIZED FEEDS* 100 ml/kg				
DATE		d d m m y y	d d m m y y	d d m m y y	d d m m y y				
Type of milk		CODES: 1=Human Milk; 2=Fortified Human Milk 3=Preterm Formula; 4=PDF							
					BOLUS		CONTINUOUS		
Start of feed					h h m m		h h m m		
					B_0 - 30 '	B_1 + 30'	C_0 - 30'	C_1 + 30'	C_2 + 3h
NIRS	$SatO_2$ %								
	rSO_2C %								
	TOI C %								
	rSO_2S %								
	TOI S %								
	CSOR N								
ECO	PSV cm/sec								
	EDV cm/sec								
	Vmean cm/sec								
	IR N								
Capillar Hemoglobine g/dL									

***enter data in cells below, irrespective of the randomized order in which feeds were given.**

Name of physician ____________   Date (dd-mm-yy) ____________   Signature of physician ____________

SPLANCHNIC OXIMETRY IN VLBW | No.4

ID: |_|_|_|_|_|_| Last name initials: |_|_|_| First name initials: |_|_|_|

GROWTH

	At birth	MEF	At achievement of 100 ml/kg/day	At randomized feeds
**WEIGHT**	|_|_|_|_| g	|_|_|_|_| g	|_|_|_|_| g	|_|_|_|_| g
**LENGTH**	|_|_| cm	|_|_| cm	|_|_| cm	|_|_| cm
**HEAD CIRCUMFERENCE**	|_|_| cm	|_|_| cm	|_|_| cm	|_|_| cm
**TYPE OF MILK**	CODES: 1=Human Milk; 2=Fortified Human Milk 3=Preterm Formula; 4=PDF		|_|	

	At full enteral feeding	At 28 days of age
**DATE**	|_|_|_|_|_|_| d d m m y y	|_|_|_|_|_|_| d d m m y y
**WEIGHT**	|_|_|_|_| g	|_|_|_|_| g
**LENGTH**	|_|_| cm	|_|_| cm
**HEAD CIRCUMFERENCE**	|_|_| cm	|_|_| cm
**TYPE OF MILK** (see above for CODES)	|_|	|_|

Name of physician   Date (dd-mm-yy)   Signature of physician

SPLANCHNIC OXIMETRY IN VLBW | No.5

ID: |_|_|_|_|_|_| Last name initials: |_|_|_| First name initials: |_|_|_|

MORBILITIES

CODES (for all, if not otherwise stated): 1=YES; 2=NO; 9=NK

PDA : |_| **IF <u>YES</u>:** 1st episode Date of diagnosis |_|_|_|_|_|_| (d d m m y y) Date of recovery |_|_|_|_|_|_| (d d m m y y)

2nd episode Date of diagnosis |_|_|_|_|_|_| (d d m m y y) Date of recovery |_|_|_|_|_|_| (d d m m y y)

Medical treatment |_| Surgical treatment |_|

Clinical sepsis |_| **IF <u>YES</u>:** 1st episode Date of diagnosis |_|_|_|_|_|_| (d d m m y y) Date of recovery |_|_|_|_|_|_| (d d m m y y)

2nd episode Date of diagnosis |_|_|_|_|_|_| (d d m m y y) Date of recovery |_|_|_|_|_|_| (d d m m y y)

IVH: |_| CODES: 1=I grade; 2=II grade; 3=III grade; 4=III grade plus; 5=NO Date of diagnosis |_|_|_|_|_|_| (d d m m y y)

PVL : |_| CODES: 1=I grade; 2=II grade; 3=III grade; 4=NO Date of diagnosis |_|_|_|_|_|_| (d d m m y y)

RDS : |_| CODES: 1=I grade; 2=II grade; 3=III grade; 4=NO

Surfactant : |_| BPD : |_|

c-PAP : |_| **IF <u>YES</u>:** 1st episode Start date |_|_|_|_|_|_| (d d m m y y) End date |_|_|_|_|_|_| (d d m m y y)

2nd episode Start date |_|_|_|_|_|_| (d d m m y y) End date |_|_|_|_|_|_| (d d m m y y)

3rd episode Start date |_|_|_|_|_|_| (d d m m y y) End date |_|_|_|_|_|_| (d d m m y y)

Intubation : |_| **IF <u>YES</u>:** 1st episode Start date |_|_|_|_|_|_| (d d m m y y) End date |_|_|_|_|_|_| (d d m m y y)

2nd episode Start date |_|_|_|_|_|_| (d d m m y y) End date |_|_|_|_|_|_| (d d m m y y)

FiO_2 >40% : |_| **IF <u>YES</u>:** 1st episode Start date |_|_|_|_|_|_| (d d m m y y) End date |_|_|_|_|_|_| (d d m m y y)

2nd episode Start date |_|_|_|_|_|_| (d d m m y y) End date |_|_|_|_|_|_| (d d m m y y)

3rd episode Start date |_|_|_|_|_|_| (d d m m y y) End date |_|_|_|_|_|_| (d d m m y y)

NEC : |_| (diagnosed within 30 days) **IF <u>YES</u>:** 1st episode Date of diagnosis |_|_|_|_|_|_| (d d m m y y) Date of recovery |_|_|_|_|_| (d d m m y)

2nd episode Date of diagnosis |_|_|_|_|_|_| (d d m m y y) Date of recovery |_|_|_|_|_| (d d m m y)

______________________ ______________________ ______________________

Name of physician Date (dd-mm-yy) Signature of physician

SPLANCHNIC OXIMETRY IN VLBW | No.6

ID: |_|_|_|_|_|_| Last name initials: |_|_|_| First name initials: |_|_|_|

FOLLOW-UP

Deviations:

Did the patient switch from the assigned nutrition modality? |_| CODES: 1=YES; 2=NO

IF YES, specify: Date: |_|_|_|_|_|_| (d d m m y y) Cause: |_| CODES : 1=Clinical decision; 2=Error; 3=Other; 9=Not known

If Other, specify: ____________________

Follow-up:

Date of last follow-up |_|_|_|_|_|_| (d d m m y y)

Status at last follow-up |_| CODES : 1=Full enteral feeding; 2=Withdrawal of consent; 3=Transfer to another hospital; 5=Drop out due to severe skin reaction; 5=Death

IF STATUS=Death, specify:

Date: |_|_|_|_|_|_| (d d m m y y) Cause: |_| CODES : 1=Sepsis; 2=Heart failure; 3=Cerebral haemorrhage, 4=Respiratory failure; 5=DIC; 6=Metabolic failure; 7=Other;9=Not known

If Other, specify: ____________________

NOTE SPACE

____

____

____

____

____

____

____

____

____

____

____

Name of physician ____________________ Date (dd-mm-yy) ____________________ Signature of physician ____________________
